# The feasibility and acceptability of trial procedures for a pragmatic randomised controlled trial of a structured physical activity intervention for people diagnosed with colorectal cancer: findings from a pilot trial of cardiac rehabilitation versus usual care (no rehabilitation) with an embedded qualitative study

**DOI:** 10.1186/s40814-016-0090-y

**Published:** 2016-08-24

**Authors:** Gill Hubbard, Ronan O’Carroll, Julie Munro, Nanette Mutrie, Sally Haw, Helen Mason, Shaun Treweek

**Affiliations:** 1 School of Health Sciences, University of Stirling, Highland Campus, Old Perth Road, Inverness, IV2 3JH UK; 2 Department of Psychology, University of Stirling, Stirling, FK9 4LA UK; 3 Centre for Health Science, School of Health Science, University of Stirling, Highland Campus, Old Perth Road, Inverness, IV2 3JH UK; 4 Moray House School of Education, Institute for Sport, Physical Education and Health Sciences, University of Edinburgh, Edinburgh, EH8 8AQ UK; 5 School of Health Science, University of Stirling, Stirling, FK9 4LA UK; 6 Helen Mason, Yunus Centre in Social Business and Health, Glasgow Caledonian University, Glasgow, G4 0BA UK; 7 Shaun Treweek, Health Services Research Unit, University of Aberdeen, Aberdeen, AB25 2ZD UK

**Keywords:** Pilot, Feasibility, Acceptability, Cardiac rehabilitation, Colorectal cancer, Physical activity

## Abstract

**Background:**

Pilot and feasibility work is conducted to evaluate the operational feasibility and acceptability of the intervention itself and the feasibility and acceptability of a trials’ protocol design. The Cardiac Rehabilitation In Bowel cancer (CRIB) study was a pilot randomised controlled trial (RCT) of cardiac rehabilitation versus usual care (no rehabilitation) for post-surgical colorectal cancer patients. A key aim of the pilot trial was to test the feasibility and acceptability of the protocol design.

**Methods:**

A pilot RCT with embedded qualitative work was conducted in three sites. Participants were randomly allocated to cardiac rehabilitation or usual care groups. Outcomes used to assess the feasibility and acceptability of key trial parameters were screening, eligibility, consent, randomisation, adverse events, retention, completion, missing data, and intervention adherence rates. Colorectal patients’ and clinicians’ perceptions and experiences of the main trial procedures were explored by interview.

**Results:**

Quantitative study. Three sites were involved. Screening, eligibility, consent, and retention rates were 79 % (156/198), 67 % (133/198), 31 % (41/133), and 93 % (38/41), respectively. Questionnaire completion rates were 97.5 % (40/41), 75 % (31/41), and 61 % (25/41) at baseline, follow-up 1, and follow-up 2, respectively. Sixty-nine percent (40) of accelerometer datasets were collected from participants; 31 % (20) were removed for not meeting wear-time validation.

Qualitative study: Thirty-eight patients and eight clinicians participated. Key themes were benefits for people with colorectal cancer attending cardiac rehabilitation, barriers for people with colorectal cancer attending cardiac rehabilitation, generic versus disease-specific rehabilitation, key concerns about including people with cancer in cardiac rehabilitation, and barriers to involvement in a study about cardiac rehabilitation.

**Conclusions:**

The study highlights where threats to internal and external validity are likely to arise in any future studies of similar structured physical activity interventions for colorectal cancer patients using similar methods being conducted in similar contexts. This study shows that there is likely to be potential recruitment bias and potential imprecision due to sub-optimal completion of outcome measures, missing data, and sub-optimal intervention adherence. Hence, strategies to manage these risks should be developed to stack the odds in favour of conducting successful future trials.

**Trial registration:**

ISRCTN63510637

## Background

Randomised controlled trials (RCTs) are used to assess the benefits and potential harm of new interventions in health care. Pilot and feasibility work is conducted to evaluate the operational feasibility and acceptability of the intervention itself and the feasibility and acceptability of a trials’ protocol design. There is little point in running large-scale (and thereby presumably expensive) trials of interventions—even those suggesting promise of effect—if these interventions are unlikely to ever see the light of day and be implemented in practice. Likewise, if trial procedures prove to be unfeasible then results about clinical- and cost-effectiveness will not be valid, thereby making it difficult for policy makers, service commissioners, and clinicians to decide whether to adopt an intervention. Conducting pilot studies to iron out methodological bias and imprecision in advance of a large-scale trial is critical if that larger trial is to become part of an evidence-base that is then used for recommending policy and changing practice.

Research funding bodies and trial methodologists recommend pilot and feasibility studies [1, 2]. If the research is novel, there are usually uncertainties regarding key trial parameters such as recruitment and loss to follow-up. These parameters are critical to ensuring that a trial is sufficiently powered to determine differences in outcomes between experimental versus control groups and to reduce risk of bias. Findings from feasibility and pilot work about trial parameters can be used to optimise the design and conduct of any subsequent large-scale trial and to judge whether it is even appropriate and ethical to proceed to such a trial.

Feasibility and pilot work may not always be necessary if similar studies exist. Trialists can assess the likelihood of success of their intervention and trial procedures by examining these studies. At the time of developing our proposal, we were aware of 12 on-going or published studies of structured physical activity interventions for people with colorectal cancer (CRC) [3–14]. These trials varied by type of intervention (e.g., exercise classes, home-based exercise prescription, counselling) and clinical endpoints (e.g., biomarkers, physical activity, quality of life). Moreover, most were small studies thereby making it difficult to draw inferences about the success of our planned trial. We therefore concluded that feasibility and pilot work of cardiac rehabilitation versus usual care (no rehabilitation) for people diagnosed with CRC was required.

The overall aims of the Cardiac Rehabilitation In Bowel cancer (CRIB) study were to assess whether using cardiac rehabilitation is a feasible and acceptable model of rehabilitation to aid the recovery of post-treatment CRC patients (i.e. examine intervention implementation potential) and to test the feasibility and acceptability of the protocol design (i.e. examine methodological standard). Thus, the purpose of the study was to assess whether it was appropriate to progress to a larger-scale trial and, if so, to optimise the design and conduct of any such trial.

A report of the feasibility and acceptability of the intervention is reported elsewhere [15]. In this manuscript, we describe and report data that directly addresses the feasibility and acceptability of trial procedures. We report screening, eligibility, consent, randomisation, adverse event, retention, completion, missing data, and intervention adherence rates. We also report reasons for exclusion, reasons for not consenting, sample characteristics, group (intervention versus control) characteristics, and distribution of the primary outcome. The findings of an embedded qualitative study about participants’ experiences of trial components are also reported. In this manuscript, we do not report site-level data (the study was conducted in three sites); however, a detailed description of site-level recruitment performance is reported elsewhere [16]. We also do not report effectiveness data because it is generally recommended that feasibility and pilot studies descriptively evaluate a trial’s feasibility, acceptability and safety rather than test the effectiveness hypotheses of the planned main large-scale trial [2, 17–19]. This is because the small amount of effect data available in feasibility and pilot studies means the degree of uncertainty is such that the chance of reaching inaccurate conclusions about intervention effect is high. Hence, robust and rigorous assessment of an intervention’s therapeutic implications must await adequately sized definitive pivotal trials [19].

## Methods

A full description of methods is available elsewhere [20]. A brief description is presented below.

### Trial methods

#### (i) Participants

People with CRC were recruited from three UK hospitals.

##### Inclusion

- ≥18 years
- Diagnosed with primary CRC
- In recovery period following CRC surgery (including those receiving adjuvant therapy such as chemotherapy and radiation therapy)

##### Exclusion

- Advanced disease
- Failure of clinical/risk assessment for cardiac rehabilitation
- Deemed unsafe to participate in exercise by cancer nurses during screening
- Severe cognitive impairment
- Unable to communicate in English (no funds available in trial for translation services)

#### (ii) Recruitment

A clinical nurse specialist assessed people admitted for surgery for CRC to determine their eligibility for the study; those eligible were given a study information sheet. After discharge from hospital, a researcher contacted people by telephone to discuss what the trial involved. If the person was interested, and ready to attend cardiac rehabilitation, a mutually convenient time for the person to meet with the researcher was arranged where eligibility was confirmed. Written consent was obtained face to face at this initial appointment. Participants who consented had baseline measures taken and were then randomised to either the intervention or control group. If the person decided not to participate in the study during the telephone call, or before giving consent at their initial appointment, a reason for declining was recorded by a researcher, if agreed by the individual.

#### (iii) Randomisation and concealment

Randomisation of individual participants to a particular trial arm (cardiac rehabilitation (intervention arm) versus non-rehabilitation (control arm) was undertaken immediately after baseline measures by a researcher using an automated online randomisation system that was managed by a UK-registered clinical trial unit. Hence, randomisation was concealed from the researcher before baseline measures but not during follow-up.

#### (iv) Treatment group allocation

##### Usual care

Patients were given a booklet by Bowel Cancer UK (a cancer charity)—‘Staying healthy after bowel cancer’.

##### Intervention

Patients were informed they would be referred to cardiac rehabilitation. A researcher completed a patient referral form and sent it on to the cardiac rehabilitation service. A member of the cardiac multi-disciplinary team (e.g. cardiac physiotherapist or nurse) then contacted the participant and invited them to attend a cardiac rehabilitation clinical/risk stratification assessment to determine whether the participant was able to safely exercise. Participants who were deemed safe to exercise were then given a date to start cardiac rehabilitation, which comprised exercises classes and cardiac-specific education sessions. Participants attended a 1-h exercise class each week over a period of 10 weeks in site 1 and 12 weeks in site 2 and attended twice weekly over a period of 6 weeks in site 3. Additionally, participants were invited along to the weekly education sessions delivered by the cardiac rehabilitation team. Session themes across the three sites included healthy lifestyle sessions (e.g. diet, physical activity, relaxation/stress management) and cardiac-specific sessions (e.g. misconceptions, medications, ‘healthy heart’).

#### (v) Outcomes to assess clinical- and cost-effectiveness and data collection

##### Outcomes

The amount of physical activity was assessed objectively using Actigraph GT3X+ accelerometer. Participants were given an accelerometer to wear around their waist during waking hours for seven consecutive days after they completed other measures (e.g. quality of life questionnaires) at baseline and similarly, for seven consecutive days after follow-up measures. There was not retrospective self-reported assessment of amount of physical activity before participants had a colorectal cancer diagnosis. The type of physical activity was assessed subjectively using the Scottish Physical Activity Questionnaire (SPAQ) to ascertain the types of activities participants engaged in [21]. Participants were asked to record the number of minutes for each day of the week spent undertaking each type of activity. *Quality of life* was assessed using the European Quality of life 5 Dimensions (EQ-5D) [22]. The Functional Assessment of Cancer Therapy-Colorectal (FACT-C) was used to measure cancer-specific quality of life [23]. *Anxiety and depression* was measured using the Hospital Anxiety and Depression Scale (HADS) [24]. *Fatigue* was measured using the Functional Assessment of Chronic Illness Therapy-Fatigue (FACIT-Fatigue) scale, [25]. *Healthcare resource use* was measured using a seven-item self-report questionnaire developed by the research team for the purposes for the study.

The following process variables were also collected: *physical activity self-efficacy* was measured using a 12-item questionnaire developed by investigators of the ActWell trial [9] and designed specifically to measure physical activity self-efficacy in the context of delivering a behaviour change intervention. *Risk perception* was measured using recommended operationalization of the concept in the context of behaviour change research [26].

##### Data collection

A researcher collected baseline measures immediately after the participant had provided written consent in order to gather data before participants allocated to the intervention group began cardiac rehabilitation. Bristol online surveys website (http://www.survey.bris.ac.uk) was used for the self-report questionnaires. A researcher read each question out and inputted the participant’s response immediately using the online system. Participants were given an accelerometer on the same day as baseline measures were collected. They were given a factsheet about wearing the device and asked to wear the device for seven consecutive days and return in the pre-paid envelope supplied to them.

The first follow-up assessment coincided with the end of the intervention delivery period (or equivalent period for participants allocated to the control group), that is, after the participant had attended the final cardiac rehabilitation class. This time scale was 10, 12, and 6 weeks for sites 1, 2, and 3, respectively. The second follow-up assessment was approximately 3 months after the participant had finished cardiac rehabilitation (or equivalent period for participants allocated to the control group). Follow-up measures were collected at the academic institution, hospital, or participant’s own home using the same procedures used to collect measures at baseline. The participants were requested to wear the Actigraph GT3X+ accelerometer for seven consecutive days for the second time.

#### (vi) Outcomes to assess feasibility and acceptability of trial parameters

Outcomes used to assess the feasibility and acceptability of key trial parameters were screening, eligibility, consent, randomisation, adverse events, retention, completion, missing data, and intervention adherence rates. Reasons for exclusion and reasons for not consenting were recorded and data to assess sample characteristics and distribution of the primary outcomes were also collected. Definitions for these key trial parameters are described below.

##### Screening rate

The screening rate was defined as the number of people with CRC who were admitted for surgery and assessed for eligibility by a clinical nurse specialist using inclusion/exclusion criteria. Demographic information was collected about all eligible patients with CRC. This included those who subsequently decided not to take part with their permission. Clinical trial software Open Clinica (https://openclinica.com) was used to enter information locally at each site allowing clinical data management for analysis.

##### Eligibility rate and reasons for exclusion

The eligibility rate was calculated in the following two ways: (a) dividing the number of people with CRC admitted for surgery by the number who met inclusion criteria and (b) dividing the number of people screened for eligibility by the number who met inclusion criteria. Data entered into OpenClinica were used for these calculations. Nurses recorded their reasons for excluding patients on a screening and recruitment form. A researcher entered these data into OpenClinica.

##### Consent rate and reasons for not participating in the study

The consent rate was calculated by dividing the number of people with CRC who met inclusion criteria and therefore eligible for the study, by the number who consented in writing to participate in the study using data entered into OpenClinica. A researcher recorded on a local site log reasons why people with CRC who met inclusion criteria and verbally consented to having their contact details given to the research team then decided not to participate in the study.

##### Randomisation rate and group characteristics

The randomisation rate was calculated by dividing the number of consenting participants by the number randomised to the cardiac rehabilitation or usual care (no rehabilitation) group. Group characteristics were compared by age, gender, diagnosis (using the Tumour, Node, and Metastases (TNM) classification system), type of treatment, and stoma.

##### Adverse events

Adverse events were recorded on a local site adverse event participant log by a researcher. Events were recorded as ‘related’ or ‘unrelated’ to the study.

##### Retention rate

The retention rate was defined as the number of participants who remained in the study, that is, the number of participants who did not formally drop out of the study using data entered into OpenClinica.

##### Completion rate

The completion rate was defined in the following two ways: (a) the number of participants who completed the self-reported questionnaires and (b) the number of participants who returned an accelerometer device that was then checked for validity by a researcher. Completion rates were calculated at baseline, T1, and follow-up.

##### Missing data

Missing data was defined as the number of participants with invalid accelerometer data. The validation parameters and cut-off points described below were chosen because they have been used in cross-sectional [27–29] and intervention studies [11] that have measured physical activity and sedentary behaviour among people with CRC. Actigraph software wear-time validation was set to meet the following criteria:

- Minimum number of valid days required = 4
- Non-wear time was set at >60 min of consecutive zeros
- Minimum number of wear hours per day required ≥10 h (600 min)

There is no international consensus about analysing accelerometer data for people with cancer [11, 30], and there have been only a handful of studies that used accelerometers to measure physical activity in people with colorectal cancer [29]. We therefore turned to the non-cancer literature for guidance including how the 54 different research teams analysed the accelerometer data from the National Health and Nutrition Examination Survey (NHANES) [31]. Research teams applied different decisions rules to analyse NHANES accelerometer data; 23 teams required a minimum of 4 days wear time, 42 teams defined non-wear time as >60 min of consecutive zeros, and 49 defined a valid day as 10 h or more of wear time.

##### Intervention adherence

Intervention adherence was measured by summing the total number of cardiac rehabilitation exercise classes attended by participants allocated to the intervention group. Data was collected from the local cardiac rehabilitation register of attendance by a researcher.

##### Sample characteristics

Sample representativeness was assessed by comparison of the characteristics of eligible consenting and not consenting patients who gave permission to have clinical and socio-demographic information about them used for the purposes of the study. Socio-demographic (e.g. age, gender) and clinical characteristics (e.g. diagnosis, treatment, temporary, or permanent stoma) were obtained, with permission, from nurses who had access to patient records.

##### Distribution of the primary outcome

Recruitment bias was assessed by examining the distribution of moderate-to-vigorous physical activity (MVPA) measured at baseline and second follow-up. MVPA was classed as >1951 counts per minute, as per Freedson [32] cut points in Actigraph software. Total MVPA was chosen because the recommended amount of physical activity for cancer patients [33] is the same as for the general public and is currently 150 min of moderate intensity physical activity or 75 min of vigorous intensity activity per week (in at least 10-min bouts) or an equivalent mix of the two [34].

#### (vii) Sample size

The aim of the study was not to provide a definitive estimate of treatment effect, so we did not have a formal sample size calculation. Rather, we estimated that we would recruit 66 participants over the 6-month period. This was based on the following:

- Number of surgical admissions across all three sites in the previous year
- Cancer clinician estimates of eligibility of surgical admissions (33 % ineligible)
- Recruitment rate from a UK trial involving physical activity and similar clinical population (estimated that approximately 33 % will consent) [35]

This recruitment period was fixed at 6 months, which is reasonable for pilot and feasibility work.

#### (viii) Analysis

Descriptive statistics were used to summarise the screening, eligibility, consent, randomisation, adverse events, retention, completion and missing data, intervention adherence rates, and sample representativeness and recruitment bias.

### Embedded qualitative study

All trial participants (i.e., people with CRC) provided informed consent at baseline to be approached by a researcher about participating in an interview. All participants were contacted by telephone and invited for interview. If they were willing to be interviewed, a mutually convenient time and place was arranged to conduct the interview. Interviews were either at the first or second follow-up depending on what was most convenient for participants. Semi-structured interviews were chosen to collect data because they allow flexibility in what sequence questions are asked and in whether and how particular areas might be followed up and developed with different interviewees [36]. Questions focused on key trial parameters such as experiences of randomization and participant burden. Two investigators analysed qualitative data, one of whom was involved in conducting the interviews and the other was the Principal Investigator. Audio-recorded interviews were transcribed verbatim and analysed thematically. The Framework approach, which is a rigorous method providing a structure within which qualitative data are organised and coded and themes identified, was used to guide the analysis [37].

### Ethical approval and research governance

National Health Service (NHS) ethics approval was provided (REC reference 13/NS/0004; IRAS project ID 121757). NHS Research Management approvals (an additional approval required in the UK for research involving NHS patients, staff, or premises) were provided by each of the three Health Boards where the study was conducted.

## Results

**Fig. 1:**
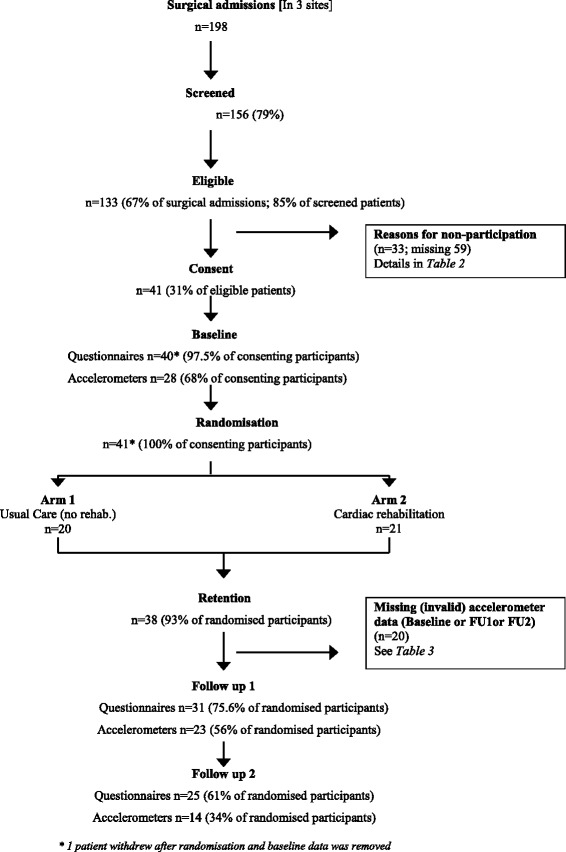
Flowchart of participants

Figure 1 shows the flow of participants throughout the trial.

### Screening rate

The screening rate was 79 %. One hundred ninety-eight people were admitted to the hospital for CRC surgery across three sites and over the 6 months recruitment period. CRC nurses assessed 156 for eligibility.

### Eligibility rate and reasons for exclusion

As previously mentioned, two calculations for eligibility were conducted. First, the eligibility rate was calculated by dividing the number of people with CRC admitted for surgery by the number who met inclusion criteria. No participants who were allocated to the intervention arm of the trial were excluded because they were deemed unsafe to exercise by the cardiac rehabilitation team. Hence, the eligibility rate represents the number of screened patients who met inclusion criteria according to the cancer clinical team. The eligibility rate was 67 %. Out of the 198 people admitted to hospital for CRC surgery, 133 met eligibility criteria.

Second, the eligibility rate was calculated by dividing the number of people screened for eligibility by the number who met inclusion criteria. The eligibility rate was 85 %. Out of the 156 people who were screened by nurses for eligibility, 133 met inclusion criteria.

Two out of three sites reported reasons for ineligibility. Table 1 shows that the main reason for excluding a patient was poor mobility. Sites 1 and 2 had a total of 116 people admitted to hospital for CRC surgery and of these, 8 (7 %) were excluded due to poor mobility and 5 (4 %) for other health reasons.

**Table 1:** Reasons for ineligibility (sites 1 and 2 only)

Reason given by nurse	Number of patients excluded	Exclusion criteria (1 to 3)
Poor mobility	8	2
Other health reason	5	2
Advanced disease	2	1
Unable to provide consent	3	3
Patient is a full-time carer	1	N/A
Unknown	4	N/A
Total	23	–

*N/A* not applicable

### Consent rate and reasons for not participating in the study

The consent rate was 31 %. Forty-one out of 133 eligible patients gave written consent. Thirty-three eligible patients who did not agree to participate in the study, however, consented to have socio-demographic and clinical information (including reasons for declining to participate) used for the purposes of the study. Reasons for non-participation are presented in Table 2. The most common reason fell into the clinical category, which included poor recovery from surgery, co-morbidity or receiving adjuvant therapy (15 out of 33, 46 %).

**Table 2:** Reasons for declining to participate (*n* = 33)

Reason	All sites
Distance/travel barriers	2 (6 %)
Return to normal activities	3 (9 %)
Clinical, e.g. poor recovery from surgery, co-morbidity	9 (28 %)
Other commitments/time	2 (6 %)
Adjuvant therapy	6 (18 %)
Study time limit	3 (9 %)
Unable to contact	1 (3 %)
Patient death	1 (3 %)
Missing (out of 33)	6 (18 %)

### Randomisation rate

Twenty-one participants were randomised to the cardiac rehabilitation group and 20 to the usual care (no rehabilitation) group. Twenty-seven men (65.9 %) and 13 (34.1 %) women were recruited to the study. The number of men allocated to the cardiac rehabilitation and usual care (non-rehabilitation) groups was 13 (61.9 %) and 14 (70 %), respectively. The mean age of participants was 66 years (SD 11.31); the youngest participant was aged 42 years and the oldest was aged 86 years. The mean age of participants allocated to the cardiac rehabilitation and usual care (no rehabilitation) groups was 67.9 (SD 11.49) and 64.2 (SD 11.10) years, respectively. There were marginal differences between participants in the intervention and control groups; for example, seven (33.3 %) participants allocated to the cardiac rehabilitation group and four (20 %) allocated to the usual care (no rehabilitation) group were classified as T3. However, it is difficult to make direct comparisons between the two groups because of missing data. Table 4 shows nine (22 %) participants had missing information about tumour size, 13 (31.7 %) had missing information about lymph nodes containing cancer cells, and 36 (87.8 %) had missing information about metastases. The most likely explanation for missing data is that Tumour, Node and Metastases (TNM) was not known at the time when diagnosis was recorded and inputted by into OpenClinica. However, there was no missing data about whether participants had colon or rectal surgery, and there was a near even split between participants allocated to the cardiac rehabilitation and usual care (no rehabilitation) groups who had colon surgery and rectal surgery. More participants allocated to the cardiac rehabilitation group compared to the usual care (no rehabilitation) group had laparoscopic surgery (*n* = 6 (28.8 %) versus *n* = 3 (15 %)), whereas more participants allocated to the cardiac rehabilitation group compared to the usual care (no rehabilitation) group had open surgery (*n* = 13 (65 %) versus *n* = 10 (47.6 %)). Four (19 %) participants allocated to the cardiac rehabilitation group had a temporary stoma or permanent stoma, whereas nine (45 %) participants allocated to the usual care (no rehabilitation) group had a temporary stoma or permanent stoma.

### Adverse event rate

No adverse events were reported in the pilot trial.

### Retention rate

The retention rate was 93 %. Three out of 41 participants formally left the study (two control and one intervention). The three that left the study were from site 3.

### Completion rate

The completion rate for questionnaires (SPAQ, FACT-C, EQ-5D, FACIT-Fatigue, HADS) at baseline, follow-up 1, and follow-up 2 was 97.5 % (20 intervention, 20 control) 75.6 % (15 intervention, 16 control), and 61 % (12 intervention and 13 control), respectively.

The completion rate for accelerometers at baseline, follow-up 1, and follow-up 2 was 68 % (14 intervention and 14 control), 56 % (11 intervention 12 control), and 34 % (6 intervention and 8 control), respectively. There was a total of 65 accelerometer device datasets across all three time-points.

### Missing accelerometer data

Twenty out of 65 (31 %) accelerometer device datasets were removed from analysis because data were invalid. Table 3 shows that the main reason for missing accelerometer data was not wearing the device (35 %).

**Table 3:** Reasons for missing accelerometer data (*n* = 20)

Reasons invalid	Intervention (*n* = 11)	Control (*n* = 9)	Total
Days worn (<4)	0	3	3 (15 %)
Hours per day (<10)	2	3	5 (25 %)
Not worn at all	5	2	7 (35 %)
Abnormal activity patterns	4	1	5 (25 %)
Total	11	9	20

### Intervention adherence

Thirteen out of 21 participants (62 %) completed the cardiac rehabilitation programme. Three participants started cardiac rehabilitation but could not complete all cardiac rehabilitation classes and five did not begin cardiac rehabilitation (38 %).

### Sample characteristics

Fifty-four out of 133 eligible patients who did not consent to participate in the study consented to have their demographic and clinical information used for the purposes of this study. Table 4 shows that there were no significant differences in age, gender, and type of surgery (colon or rectal) between consenting and non-consenting eligible patients but suggests that people with metastatic disease (T4 and N1/N2 classification), having open surgery, and with a stoma are more likely not to participate.

**Table 4:** Characteristics of consenting and not consenting eligible patients

	Not consenting *N* (%) total *N* = 54	Consenting *N* (%) total *N* = 41
Age (years)		
*N*	54	41
Missing	0	0
Mean	65.6	66.0
SD	13.81	11.31
Median	65.5	67.0
Sex		
Male	39 (72.2)	27 (65.9)
Female	15 (27.8)	14 (34.1)
Primary tumour^a^		
Missing	13 (24.1)	9 (22.0)
T0	2 (3.7)	1 (2.4)
T1	1 (1.9)	3 (7.3)
T2	8 (14.8)	12 (29.3)
T3	20 (37.0)	11 (26.8)
T4	10 (18.5)	5 (12.2)
Regional lymph node^a^		
Missing	19 (35.)	13 (31.7)
Nx	1 (1.9)	0
N0	21 (38.9)	22 (53.7)
N1	13 (24.1)	6 (14.6)
Distant metastasis^a^		
Missing	48 (88.9)	36 (87.8)
M0	5 (9.3)	5 (12.2)
M1	1 (1.9)	0
Colon surgery		
Yes	33 (61.1)	25 (61)
No	21 (38.9)	16 (39)
Rectal surgery		
Yes	19 (35.2)	16 (39)
No	35 (64.8)	25 (61)
Laparoscopic surgery		
Missing	1 (1.9)	0 (0)
No	37 (68.5)	32 (78)
Yes	16 (29.6)	9 (22)
Open surgery		
Missing	1 (1.9)	0
No	21 (38.9)	18 (43.9)
Yes	32 (59.3)	23 (56.1)
Temporary stoma		
Missing	3 (5.6)	0
No	39 (72.2)	35 (85.4)
Yes	12 (22.2)	6 (14.6)
Permanent stoma		
Missing	3 (5.6)	0 (0)
No	45 (79.6)	34 (82.9)
Yes	8 (14.8)	7 (17.1)
Chemotherapy		
Missing	11 (20.4)	7 (17.1)
No	37 (68.5)	27 (65.9)
Yes	6 (11.1)	7 (17.1)
Radiotherapy		
Missing	10 (18.5 %)	7 (17.1)
No	36 (66.7 %)	29 (70.7)
Yes	8 (14.8 %)	5 (12.2)
Other treatment		
Missing	4 (7.4 %)	3 (7.3)
No	49 (90.7 %)	35 (85.4)
Yes	1 (1.9 %)	3 (7.3)

^a^TNM stands for Tumour, Node, and Metastases. This staging system describes the size of a primary tumour (T), whether any lymph nodes contain cancer cells (N), and whether the cancer has spread to another part of the body (M)

### Distribution of the primary outcome

Minutes of MVPA per day was calculated by dividing the total score by number of days worn. Table 5 shows that participants were meeting or not far off meeting the recommended level for MVPA (i.e., 30 min a day). MVPA per day scores were skewed towards lower scores at baseline but normally distributed at 3-month follow-up.

**Table 5:** Minutes per day of MVPA

Variable	Intervention	Control	Total
Sum of moderate–vigorous time (minutes per day)			
*N*	14	14	28
Missing	0	0	0
Mean	21.1	29.0	25.1
SD	11.68	35.90	26.50
95 % LCL	14.40	8.27	14.80
95 % UCL	27.89	49.72	35.34
Median	20.6	10.5	17.8
Sum of moderate–vigorous time (minutes per day)			
3-month follow-up			
*N*	6	8	14
Missing	0	0	0
Mean	22.5	54.5	40.8
SD	17.41	28.34	28.63
95 % LCL	4.27	30.85	24.30
95 % UCL	40.81	78.24	57.36
Median	23.9	56.3	35.6
3-months follow-up minus baseline			
*N*	6	7	13
Missing	0	0	0
Mean	1.3	10.5	6.2
SD	15.04	28.37	22.79
95 % LCL	−14.51	−15.74	−7.53
95 % UCL	17.06	36.73	20.01
Median	0.3	7.7	4.2

### Findings from the qualitative interviews

#### (i) Themes

Twenty-two participants (12 cardiac rehabilitation group and 10 usual care (no rehabilitation group)) agreed to an interview. Unsurprisingly, themes closely matched the focus of the interview, which included questions about trial procedures. The main theme was ‘barriers to involvement in a study about cardiac rehabilitation’ with the following sub-themes: randomisation, study information, and participant burden (questionnaires and accelerometers). Table 6 presents participant quotations, which were selected by the research team to illustrate each sub-theme.

**Table 6:** Participant quotations for each sub-theme

Sub-themes	Quotations^a^
Randomisation	“Fine, I had no feelings one way or another [about being allocated to cardiac rehabilitation or usual care (no rehabilitation) group]. I was quite happy to participate one way or t’other.” (site 1 23 usual care)
	Investigator: Were you a little bit disappointed [not being allocated to the cardiac rehabilitation group]?
	Participant: I was because I thought the exercise might help me.” (site 2 09 usual care)
Study information	“I got a big form with more information but I haven’t read it [laughs].” (site 2 17 usual care)
	Investigator: “Do you feel that all the information you were given was clear?
	Participant: Yes I’m sure it was… I can’t remember reading the booklet. I think it was just verbal but maybe I should have read the booklet [laughs].” (site 2 09 usual care)
	Investigator: “Was it an appropriate time to discuss the study?
	Participant: Yeah.
	Investigator: There was nothing inappropriate about the timing or insensitive?
	Participant: Not for me anyway.” (site 2 17 usual care)
Participant burden: questionnaires	“Oh they’re fair, the questions are fair, yes, and eh, I mean, there’s nothing that I’m stumbling to answer, you know, it’s very simple and eh straightforward.” (site 1 02 cardiac rehabilitation)
	“I think they’re quite long.” (site 1 13 cardiac rehabilitation)
	“A wee bit long, a wee bit long, I'm saying eh, and, no, eh aye seemed tae be getting the same question, again and again.” (site 2 02 cardiac rehabilitation)
Participant burden: accelerometers	“Easy to use. I think I wore it quite diligently.” (site 1 19 usual care)
	“I realised you had to do it [wear accelerometer] but I was putting up with so much with stitches round my rear end, stitches from here to there like top to bottom on my front, plus the [stoma] bag and all the rest.” (site 3 02 usual care)

^a^Identifiers are the site (1 to 3), unique participant number and whether the participant was allocated to the cardiac rehabilitation or usual care (no rehabilitation) group

#### (ii) Randomisation

For some participants, randomisation did not seem to be a major barrier to study participation because they did not mind which group they were allocated to. Nevertheless, some participants expressed disappointment being allocated to the usual care (no rehabilitation) group. Some participants were not clear how randomisation worked and its implications. Further, the impression given was that some participants allocated to the usual care (no rehabilitation) group felt abandoned.

#### (iii) Study information

When participants were asked about study information, most gave a perfunctory response. They either briefly replied that the information was clear or they gave the impression that they could not remember what they had been given. Thus, although study information was not necessarily a barrier to participation, neither did it appear to promote participation. None of the participants raised any concerns about being approached about the study when they were on the surgical ward, either waiting for surgery or recovering from surgery and waiting to be discharged.

#### (iv) Participant burden

Some participants did not have any problems with the questionnaire, whereas others felt it was perhaps too long and repetitive, taking up to 35 min to complete in some cases. Nevertheless, the overall impression given was that the questionnaire was not a major burden for participants to complete. Some participants commented on the length of the questionnaire. Other participants commented on question repetition.

Some participants reported no problems wearing the accelerometer. Nevertheless, other participants reported problems wearing the accelerometer. The device proved particularly troublesome to wear for those who had a stoma or abdominal wound problems. Some participants appeared to be self-conscious when wearing the device.

## Discussion

The success of a large-scale multi-centre pragmatic trial of an intervention should not be judged on whether or not the results show that an intervention is successful or not. A biased and imprecise pragmatic trial with results that show an intervention is effective is not a useful trial. According to Consolidated Standards of Reporting Trials (CONSORT) guidance, interpretation of the results (internal validity) and generalizability (external validity) of the trial results are two key factors that can be used to assess whether a trial is useful and hence, successful [38]. Pilot and feasibility studies may help researchers to predict whether any future trial is likely to be useful, and data from pilot and feasibility studies can be used to improve trial procedures to maximise the chances of any future trial being successful.

The screening rate of potential participants can be used to assess if those recruited to a study are likely to be representative or typical of the target population. If a large number of people are not screened for eligibility then this may indicate potential recruitment bias. Only two published studies involving physical activity intervention for CRC patients have included screening rates [11, 14]. In the CRIB study, the majority of the target population were screened. Thus, based on the screening rate of the CRIB study, we can estimate that participants in a future large-scale will be typical of the target population.

Another indicator that can be used to assess if participants are representative of the target population and therefore how confident we can be in generalising study results to the target population is by comparing the characteristics of eligible consenting and not consenting patients. In the CRIB study, we found that there were no meaningful differences in age, gender, and type of surgery (colon or rectal) between consenting and non-consenting eligible patients but people with metastatic disease (T4 and N1 classification), having open surgery, and with a stoma, were more likely not to consent. Our qualitative study highlighted that a stoma was problematic for some participants. Additionally, people with a stoma may be embarrassed [16]. Future trials of structured physical activity interventions should therefore consider ways of addressing problems of participation associated with a stoma. Moreover, if a sample is to be representative and typical of the target population then strategies to support people who have had open surgery to participate in a structured physical activity intervention or are living with metastatic disease may need to be implemented.

Examining the distribution of participants on outcomes can be used to assess potential recruitment bias. In the CRIB study, at baseline, most participants were averaging 30 min of MVPA a day and therefore meeting the recommended guidelines for physical activity for cancer patients [33] and the general population [34]. However, studies have shown that most CRC patients [39–42] and members of the general population [43] are not meeting these targets. Thus, in the CRIB study, there was recruitment bias in favour of physically active CRC patients. This has important implications for those who are making decisions about treatment options because there seems little clinical value in providing a service to these patients because they are likely to be obtaining health benefits associated with post-diagnosis physical activity (unless of course, there is a dose effect for physical activity and the intervention is designed to support people already meeting recommended guidelines become even more active). Given that most patients in the CRIB study were physically active, it is likely that extra efforts will have to be made to recruit people who are less active. Introducing motivational strategies, such as providing information about the benefits of being active, may help recruitment because according to behavioural change theory, internalisation of the value (the benefits) of the outcomes of physical activity is likely to lead to greater persistence in being physically active [44]. Moreover, other studies have excluded people with CRC who are already meeting recommended guidelines for physical activity, suggesting that it is possible to recruit inactive CRC patients [6, 11].

Eligibility rates can be used to assess if those recruited to a study are likely to be representative of a target population and thereby the generalizability of the study. If most of the total population are eligible, then we can conclude that the study is highly representative of the target population. In the CRIB study, the eligibility rate was 67 %. However, most studies of structured physical activity interventions with the exception of Lee et al. (2013) [14] and Pinto et al. (2013) [11] do not report the total population from which the sample was drawn and calculate the eligibility rate as the proportion of screened patients who met inclusion criteria [3–14]. The problem with this approach however is that there is no way of knowing how many patients were excluded from the study because they were not screened. Future studies should therefore consider reporting the total population and the screening rate.

In pragmatic trials, where the aim is to assist decision makers about treatment options, the principle of equitable healthcare [45] is likely to be taken into consideration and should therefore be examined in feasibility and pilot studies. Poor mobility and other health problems were the most common reasons nurses gave for excluding patients in the CRIB study. Yet, some people with poor mobility and health problems may benefit from a structured physical activity intervention and should therefore be included. The CHALLENGE trial has recently reported that staff did not approach people who ‘do not look like an exerciser’ [46]. Hence, research teams should make an effort to ensure that all staff define eligibility criteria appropriately. If exclusion from trials is a proxy for exclusion from interventions should they be implemented as part of routine healthcare, then the principle of equitable healthcare seems therefore at risk for structured physical activity interventions for CRC patients. Induction and training of recruiters about the benefits of physical activity and contraindications for exercise may therefore be particularly important to ensure that structured physical activity studies and interventions, if implemented, are equitable. Yet, studies also need to make sure that participants are safe to exercise. Some studies (including the CRIB study) relied on clinician judgement to decide if it was safe for a patient to exercise [11, 13], and other studies used forms of assessment such as fitness tests [12, 14] or disease indices [7]. Adverse events are also an indicator of safety. In the CRIB pilot trial, no adverse events were reported.

The randomization process can lead to potential bias if methods used to allocate participants to the different arms of the trial do not create a situation where participants have a 50:50 chance of being allocated to one group or the other. In the CRIB study, an electronic random allocation sequence generator managed by a registered UK clinical trial unit was used thereby making it unlikely that the randomisation process was biased. In the CRIB study, we compared the characteristics of participants allocated to the two arms of the trial to assess for any bias. Missing data about diagnosis meant that we were not able to make all comparisons but there were differences between groups on some variables, such as more patients in the usual care (no rehabilitation) group having a stoma. These differences may impact on outcomes, and therefore, it is important that these data are reported. Moreover, while allocation to the two arms of the trial was concealed from the researcher before baseline measures, it was not concealed during follow-up measures, which could introduce bias..

To facilitate interpretation of the results of a trial, CONSORT guidelines recommend understanding factors that suggest potential imprecision [38]. A low consent rate could indicate potential imprecision because a study may be underpowered. Underpowered studies limit the ability to draw conclusions about the effect of an intervention on health outcomes and are more likely to go unpublished or report statistically non-significant results. Poor recruitment for physical activity interventions increases the chance of the trial being abandoned, with potentially important clinical effects of that intervention not getting shared or reported [43–45]. The CRIB study’s consent rate was 31 %, which is higher than some studies that report 10 % [6] and 12 % [14] but lower than other studies that report 78 % [13] and 70 % [11] consent rates. Understanding reasons for non-participation is therefore important because this information can be used to improve consent rates in future studies and, in turn, minimise one of the threats to precision of a trial. However, in the CRIB study, only 33 of eligible participants who did not consent to participate in the study agreed to give a reason why they did not wish to participate and twice as many (*n* = 65) did not agree to give a reason. This may be because researchers did not clearly explain the importance to research of collecting such information. In the CRIB study and other studies of structured physical activity interventions for CRC patients, ‘medical conditions’ and ‘not interested’ are the most common reasons given for eligible participants not consenting [3, 5, 11–14]. These factors may also explain why CRC patients drop out of intervention trials. Studies promoting change in diet and physical activity in individuals with a diagnosis of colorectal cancer reported health concerns, personal reasons, and inability to commit as reasons for participant drop-out [47]. Encouraging eligible people to participate in a physical activity trial however will be challenging and strategies to motivate this group to participate in a clinical trial and maintain engagement in physical activity interventions should be developed. Strategies are likely to comprise those that are relevant to clinical trial participation in general such as improving communication between clinicians and patients with cancer about research [48] as well as strategies that directly address barriers to physical activity participation in people with CRC such as fatigue [49].

Another trial parameter that indicates potential imprecision is loss of participants to a study, which may be due to a combination of factors, including participants formally dropping out of the study (retention rate), failing to complete outcome measures (completion rate), or failing to provide valid data (missing data). Loss of participants during trial follow-up can introduce bias and reduce power, thereby affecting the generalisability, validity and reliability of results [50]. Thus, information about retention and completion rates and missing data is important for assessing potential bias and imprecision. It has been estimated that a 20 % loss can threaten trial validity [51]. Some missing data can be dealt with statistically and therefore may be regarded as less of a problem than poor retention and completion; nevertheless, the risk of bias and imprecision due to missing data can remain [52] and therefore should be reported alongside other rates. Retention was excellent in the CRIB study, but the pilot trial suggests that completion rates compared to other similar studies were below par. The self-report questionnaire completion rate at second follow-up in the CRIB study was 61 %, compared to, for example, 78.5 % [13] and 88 % [11] completion rates in other studies of structured physical activity interventions for CRC patients. Our qualitative study suggests that participant burden is a factor and that lengthy or repetitive questionnaires may impact completion rates. A recent systematic review of 38 randomised retention trials evaluating six broad types of strategies to increase questionnaire response and retention in randomised trials concluded that no strategy had a clear impact on increasing the number of participants returning to sites for follow-up but found that the following strategies may improve questionnaire response: addition of monetary incentives for return of postal questionnaires, recorded delivery of questionnaires, and a ‘package’ of postal communication strategies with reminder letters [53]. The qualitative interviews also highlighted that some participants allocated to the control group felt abandoned, which may also have influenced completion rates [54]. Further research about the impact of strategies to reduce loss of participants to a study is therefore required.

Our primary outcome was objective measurement of physical activity using accelerometer. Hence, completion and missing data rates for this endpoint is of particular concern. Objective measures of physical activity have been increasingly used to overcome limitations of self-report measures. Research conducted among the general population suggests that self-reported measures of physical activity are inaccurate when compared with objective measurement from devices such as accelerometers [55–57]^.^ Despite the advantages of obtaining an objective measure of physical activity, there are few guidelines for using accelerometers in research [58, 59] and little guidance on improving participant compliance [60]. Strategies to optimise completion rates and analyses of accelerometer data include the use of log sheets for participants to record dates/times that the device is worn, wear-time algorithms, and use of ‘conventional’ cutpoints and number of hours and days to determine [61]. Nonetheless, as a recent review of literature about accelerometers concluded, questions still remain about their validity [62]. In the CRIB study, completion rates fell from 68 % at baseline to 34 % at second follow-up. In addition, 31 % of accelerometer datasets were invalid, and this was mainly because participants did not wear the device. As our qualitative study shows, wearing the accelerometer may be bothersome for patients who have a stoma or wounds from surgery and these factors should be taken into consideration when selecting instruments to measure outcomes. Recommended approaches for improving compliance include a daily monitoring log filled out by participants, reminder phone calls, adequate education about the monitor and its proper wear, and identification of potential barriers to wearing with each participant [63]. These strategies should be tested in studies of a similar context involving CRC patients.

A critical component to the success of physical activity intervention trials is achieving high levels of adherence (i.e. the extent to which the intervention group performs the physical activity prescription). This is primarily because adherence is related to a trial’s health outcomes [64, 65]. Moreover, low adherence increases the risk of policy and service commissioners rejecting interventions that may actually be effective if adherence levels were optimal. Addressing adherence barriers is therefore crucial. In the CRIB study, 62 % of participants randomised to the intervention group completed cardiac rehabilitation and the main reason for either not starting cardiac rehabilitation or stopping was poor physical health. Other studies have reported 90 % [6] and 72 % [13] adherence rates, suggesting that it is possible to achieve high rates of adherence than the CRIB study achieved with this clinical population. Nevertheless, our adherence rates may be more realistic of adherence levels under NHS conditions and therefore possibly of more interest and use for service commissioners. Indeed, one of the strengths of pragmatic as opposed to explanatory trials is that it is more likely to mirror what would happen in normal practice [38].

### Limitations

This pilot trial was conducted in only three sites and recruited only 41 patients, out of a predicted 66 based on surgical admissions. A note of caution is therefore required when using these study data for estimating recruitment rates etc. for any future large-scale multi-centre trial. Some data were missing, thereby making it difficult to accurately assess potential bias, imprecision, and generalizability. There is also further work needed for measuring physical activity accurately in clinical populations. Measuring sedentary time accurately and avoiding confusion with non-wear time is a consideration with the current methods. Nevertheless, the study highlights where threats to internal and external validity are likely to arise in any future studies of comparable structured physical activity interventions for colorectal cancer patients using methods being conducted in similar contexts.

## Conclusions

Feasibility and pilot studies are useful in highlighting where potential threats to the internal and external validity of a large-scale multi-centre are likely to occur. Thus, key trial parameters should be precisely described in a pilot study and should include screening rate, eligibility rate and reasons for exclusion, consent rate and reasons for not consenting, randomization procedure and a comparison of intervention and control group characteristics, adverse events, retention rates, completion rate, missing data, sample characteristics, distribution of the primary outcomes, and intervention adherence. The CRIB study suggests that there is likely to be potential recruitment bias in this clinical population. There is also likely to be potential imprecision due to sub-optimal completion of outcome measures, missing data, and sub-optimal intervention adherence. Hence, strategies to manage these risks should be developed to stack the odds in favour of conducting a useful multi-centre trial. These findings are applicable to interventions of similar methodology, and in comparable clinical populations.

## Abbreviations

CRC, colorectal cancer; CRIB, cardiac rehabilitation in bowel cancer study; SPAQ, Scottish Physical Activity Questionnaire; MVPA, moderate-to-vigorous physical activity; TNM, Tumour, Node, and Metastases; FACT-C, Functional Assessment of Cancer Therapy-Colorectal; EQ-5D, European Quality of life 5 Dimensions; FACIT-Fatigue, Functional Assessment of Chronic Illness Therapy-Fatigue; HADS, Hospital Anxiety and Depression Scale; CONSORT, Consolidated Standards of Reporting Trials
